# Usage Trends of Student Counseling Rooms at Juntendo University's Hongo and Ochanomizu Campuses and at Other Universities in Japan: A Perspective Review

**DOI:** 10.14789/ejmj.JMJ26-0023-P

**Published:** 2026-06-18

**Authors:** YOSHIHIDE TAKESHITA, SACHI YAO, TOMOKO KURATA, TOMOKO SHIMOKAWA, NARIMASA KATSUTA, YUJI NISHIZAKI, TOSHIHIRO MITA, YUUICHI TOMIKI

**Affiliations:** 1 Division of Medical Education, Juntendo University Faculty of Medicine, Tokyo, Japan; 1 Division of Medical Education, Juntendo University Faculty of Medicine, Tokyo, Japan; 2 Student Counseling Rooms at Juntendo University Hongo and Ochanomizu Campuses, Tokyo, Japan; 2 Student Counseling Rooms at Juntendo University Hongo and Ochanomizu Campuses, Tokyo, Japan; 3 Department of Safety and Health Promotion, Juntendo University, Tokyo, Japan; 3 Department of Safety and Health Promotion, Juntendo University, Tokyo, Japan; 4 Department of Tropical Medicine and Parasitology, School of Medicine, Juntendo University, Tokyo, Japan; 4 Department of Tropical Medicine and Parasitology, School of Medicine, Juntendo University, Tokyo, Japan

**Keywords:** mental health, university students, counseling services, medical education, depression

## Abstract

Juntendo University has three faculties and three graduate schools on its Hongo and Ochanomizu campuses, which collectively serve over 3,000 students. In 2019, the university established a Student Counseling Center to provide psychological support for students from diverse backgrounds. Following the expansion of services, including the addition of psychologists and psychiatrists, both the number of operating days and the volume of consultations increased markedly, particularly from 2023 onward. This trend mirrors patterns reported at other universities in Japan and reflects the growing global recognition of the importance of student mental health support. In collaboration with student affairs departments across faculties, we conduct regular awareness activities to promote mental health and encourage service utilization. Despite these efforts, several challenges remain, including limited awareness of the center among students, space and accessibility constraints affecting the counseling environment, and the need to enhance support for the mental well-being of university students.

## Introduction

**Figure 1:**
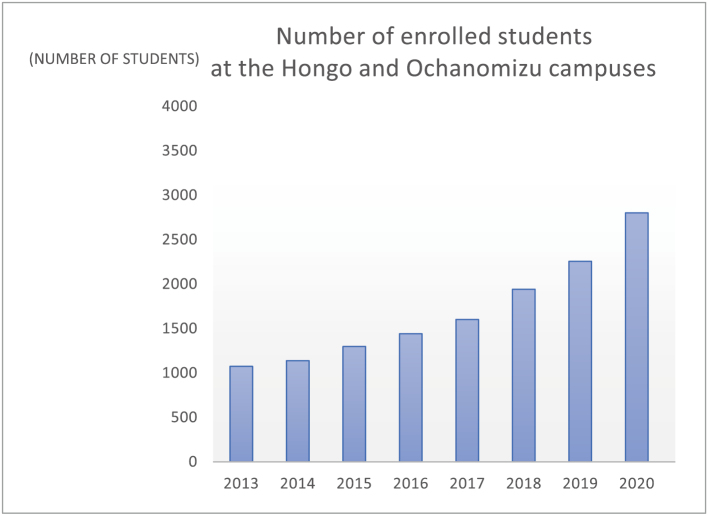
Number of enrolled students at the Hongo and Ochanomizu campuses

Juntendo University, founded in 1838, has expanded to include 9 undergraduate schools and 6 graduate schools^1)^. The number of students at the Hongo and Ochanomizu campuses has increased significantly since 2015 (Figure 1), with more than 3,000 students enrolled in the School of Medicine, School of International Liberal Studies, School of Health and Medical Sciences, and the graduate schools^2)^.

Some of these students have taken leave of absence due to mental health issues. When considering mental health problems among such students, it is often assumed that they experience reactive depression due to anxieties about interpersonal relationships at school or academic performance. However, this issue is more complex, as it also occurs in individuals with a background of developmental disorders or mental illnesses such as schizophrenia. Furthermore, even in the absence of a history of such mental illnesses, underlying factors include anxieties about obtaining qualifications and finding employment after graduation, the rapid development of information technology, major changes in the social environment in modern Japan, and financial problems for the individual student or family due to the widening of the so-called "unequal society^3)^". In many cases, symptoms cannot be expected to improve by simply addressing problems within the university, and it is difficult for university faculty and staff to handle the situation alone; a response from staff with broader perspectives and specialized knowledge is required. To address these concerns, the university encourages early psychiatric outpatient consultations and has established a student counseling center on campus to provide structured psychological support.

As mentioned above, in recent years, global attention has been drawn to the mental health of young people following rapid changes such as increased use of social networking services (SNS), the diversification of values, and the widening of socioeconomic disparities^4-6)^. Globally, suicide remains a leading cause of death among young people, prompting serious concern from the World Health Organization (WHO). A domestic survey conducted by the Ministry of Health, Labor, and Welfare (MHLW) also reported a sharp increase in mental illness among those under 24 over the 18 years leading up to 2020, highlighting the need for targeted interventions^7, 8)^.

The COVID-19 pandemic, which began in early 2020, increased young people’s need for psychological support. Prolonged lockdowns and restrictions have exacerbated social isolation and are associated with increased suicide risk, self-harm, and symptoms of depression and anxiety^9-12)^. Decreased physical activity and increased social media use have also been reported during this period.

A survey on suicide incidence conducted by MHLW^13)^ also found that the suicide rate among young people has been increasing since 2020 and is expected to remain high even after pandemic-related restrictions were relaxed in 2023. These findings suggest that a decline in interpersonal relationships may contribute to the deterioration of young people's mental health.

At Juntendo University, the shift to distance learning and modifications to clinical and practical training substantially affected student life, coinciding with an increase in consultations from students at the Student Counseling Center.

Consequently, counseling staff have been required to provide more individualized and comprehensive support compared with the pre-pandemic period.

Therefore, this review aims to describe the utilization patterns and consultation content of the Student Counseling Center at Juntendo University since its establishment in 2019 and identify ongoing and future operational challenges. By examining institutional data, this perspective review seeks to contribute to the broader discussion on strengthening mental health support systems within university settings in Japan.

## Background of the establishment

**Figure 2:**
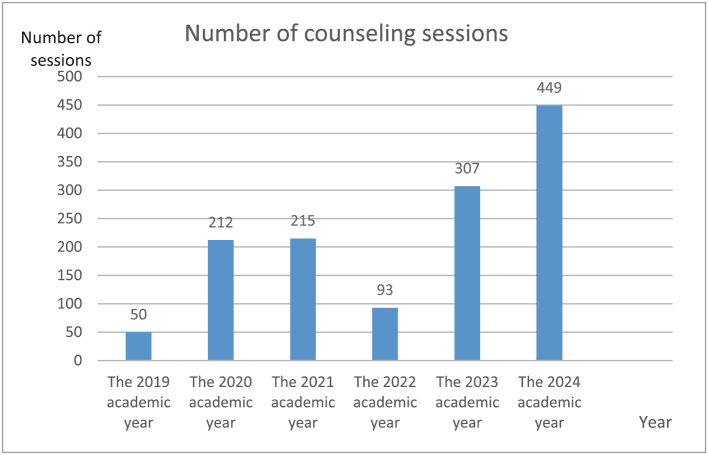
Trends in the number of consultations by fiscal year

With the continued growth of the student population at Juntendo University, the number of students reporting anxiety and depression has also increased. These concerns are associated with multiple stressors, including living alone, difficulty adjusting to university life, financial pressures, interpersonal relationships, and career-related issues. Furthermore, with the increasing number of international students, some students take leaves of absence or drop out due to depression related to difficulties adjusting to life and studying in Japan. International literature consistently provides strong evidence of the effectiveness of psychological support for university students. Counseling interventions have been shown not only to improve mental health outcomes but also to positively influence health- related behaviors, including dietary habits and smoking^14-16)^. Students with attention-deficit hyperactivity disorder (ADHD) often experience depressive symptoms due to their condition^17)^. This highlights the importance of psychological support. Juntendo University established a Student Counseling Center on its Hongo and Ochanomizu campuses in October 2019. Since the center expanded its opening hours to 5 days a week in July 2023, the number of consultations has increased significantly (Figure 2). In October of the same year, a psychiatrist was assigned to care for students requiring medication, and an outpatient consultation desk dedicated to students was opened.

## Public relations activities for the counseling center and trends in consultation volumes

Although mental health issues among university students are widely acknowledged, stigma surrounding mental illness persists both in Japan and internationally, often discouraging students from seeking psychiatric care or psychological support^18, 19)^.

**Figure 3:**
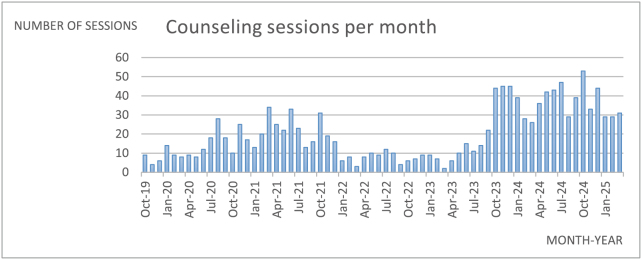
Trends in the number of consultations by month from fiscal 2019 to fiscal 2024

We believe it is necessary to promote accurate understanding and regularly consult with student affairs staff in each faculty to develop strategies for raising awareness of the Counseling Center. We also conduct awareness activities, including distributing pamphlets^20)^ and displaying posters in classrooms during mental health classes each semester. Following the intensification of these outreach efforts, consultation numbers increased from October 2023 onward. Consultation volume shows seasonal variation, with peaks observed between May and July and again from October to early December. The monthly number of consultations remains around 30 (Figure 3) but tends to decrease during long holidays and examination periods.

## Student counseling center operations

**Figure 4:**
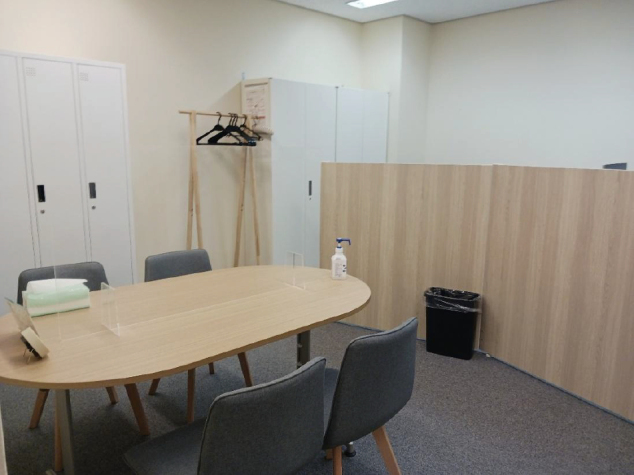
Interior of the consultation room

The Student Counseling Center is located approximately a 10-minute walk from the Student Lecture Building. Counseling sessions are conducted by appointment and last 45 minutes. Written consent is obtained before consultation regarding the sharing of personal information and communication with hospitals in case of emergencies. The counseling space is separated from the administrative office area by a partition (Figure 4); when appointment times overlap, an additional private room is utilized to ensure confidentiality. During the 2020 pandemic, computer- and telephone-based counseling was recommended^21)^. However, based on various discussions^22-24)^, face-to-face counseling has been the standard since 2024, except in cases of illness or internships. We also regularly exchange opinions with student affairs representatives from each faculty regarding maintaining and promoting student mental health.

## Consultation content and severity at our university’s counseling center

From 2023 onward, the primary focus of consultations has been mental health concerns and academic difficulties. However, consultations often cover multiple topics and have various underlying causes. For example, contributors to students' mental symptoms include serious conflicts within the family or among friends, separation from a romantic partner, or a significant discrepancy between one's expectations of university life and reality. Due to restrictions on academic and extracurricular activities during the pandemic, many students struggled to build friendships in the initial stages, and these difficulties became apparent later. In some cases, students use the center to restore disrupted daily routines or seek advice from artificial intelligence tools due to relationship problems on social media or a lack of people to talk to. The types of consultations have increased consistent with changing social conditions and were uncommon 10 years ago. Staff are now required to keep up with the rapidly changing social context and develop deeper insights into it. Until recently, the center had primarily been providing general psychotherapy, but since 2023, staff skilled in clinical movement therapy have been providing specialized treatment when necessary, and there have been cases for which symptoms have gone into remission through a combination of these approaches with drug therapy. Students suspected of having developmental disorders often exhibit maladjustment to university life due to their characteristics and sometimes utilize the center at the recommendation of faculty members. The center's staff members have many years of experience in education and often support such students suspected of having developmental disorders by guiding them through points to be mindful of in daily life and strategies for smoothly navigating interpersonal relationships. Further, some cases involve symptoms associated with pathological experiences of schizophrenia or problems related to addiction. Additionally, there has been an increase in the number of students who are referred from the center to outpatient clinics to receive pharmacotherapy. In addition to formulating treatment plans for mental illnesses, the Counseling Center collaborates with faculty members to consider reasonable accommodations when developmental characteristics affect academic performance, based on psychological assessments conducted at affiliated hospitals. Addiction among university students is a major concern internationally^25, 26)^, but the effectiveness of psychological interventions has also been demonstrated^27)^. At our university, consultations related to addictive behaviors, including alcohol use, are also observed, and appropriate support is provided in collaboration with faculty and staff. Students exhibiting psychotic symptoms requiring hospitalization are also referred to the psychiatry outpatient department. A psychiatrist joined the department in October 2023, enabling a more rapid response.

**Figure 5:**
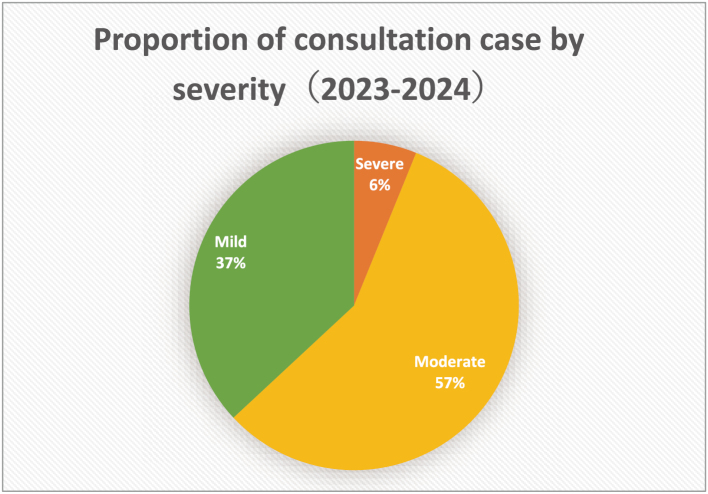
Proportion of counseling sessions by severity (2023-2024)

From October 2023 onward, each consultation case is classified as mild (treatment is possible within the center), moderate (requiring medication), or severe (requiring hospitalization). Mild cases account for 49%, moderate cases for 45%, and severe cases for 6% (Figure 5). For severe cases, hospitalization may be considered with parental involvement.

## Discussion

**Figure 6:**
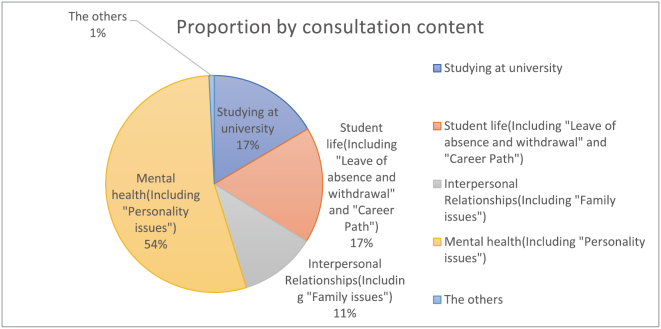
Proportion by consultation content

In recent years, international organizations, including WHO, have emphasized the rising prevalence of mental health disorders among young people and the need for coordinated societal responses. This is partly because young people are inherently at high risk for mental illness, as well as their frequent exposure to adverse events, such as pandemics and conflicts like the war in Ukraine. These events have been associated with increased anxiety and depressive symptoms^28, 29)^. Among university students, suicide has been linked to interpersonal difficulties, academic stress, and concerns related to employment and career planning. Early social and psychological interventions to address these risks have been shown to be effective. Burnout syndrome is particularly prevalent among medical students, both in Japan and overseas^30-32)^. Early preventive intervention is crucial^33, 34)^. In the present review, we compared consultation patterns at our university's Counseling Center with a domestic report examining suicide-related factors among university students^12)^. While a direct comparison is difficult, Figure 6 indicates that, when compared with national statistics, our university's consultation rates show no significant differences in the proportion of consultations related to general university life, including interpersonal relationships, academics, finances, job hunting, and careers. By contrast, there were slight differences in the proportion of health-related consultations, including depression. However, national statistics show a high proportion of cases classified as "unknown cause," so we believe the difference is minimal when these are included.

We also compared utilization patterns at our center with reports from other universities and observed similar bimodal trends in consultation frequency^35, 36)^. At first glance, the number of consultations appears to decrease during university breaks, suggesting that students' mental health symptoms improve during the holidays. However, national suicide statistics show that the number of suicides between January and March remains high^37)^, with little seasonal variation. This suggests that the decrease in consultations during this period may be due to reduced utilization rather than an actual improvement in psychological symptoms. Most campus counseling centers, including ours, remain open during breaks, yet experience a consistent decline in user numbers. Furthermore, reports indicate that many suicides occur without prior psychiatric treatment, supporting the view that there is a latent demand for psychological support throughout the year.

Stigma surrounding psychiatric and psychological services remains strong in general. This stigma tends to discourage students with mental illnesses who require treatment from using support services^38)^. Addressing these issues remains an urgent priority.

Educational outreach to improve mental health literacy is essential, but environmental and structural factors such as the location, accessibility, and comfort of counseling facilities also play important roles. There were a few differences between our university and other Japanese universities in terms of systems, such as counseling time (usually 40-60 min) and the number of psychologists assigned. However, at many universities, counseling rooms are located in prominent locations on campus^39-41)^. These factors contribute to increased awareness and accessibility. We must also strive to make similar improvements, as outlined below.

## Future challenges for the counseling center

The Counseling Center faces several challenges. First, informing students and faculty who are unaware of how to use the Counseling Center is a challenge. As part of the Counseling Center's public relations activities, staff members promote the center during on-campus lectures. We also advertise in classrooms and administrative offices frequently visited by students and regularly hold meetings with student representatives from each faculty to provide information on how to use the center. However, further publicity is needed.

Second, location is an issue. The Counseling Center is approximately a 5-10-minute walk from the lecture building, making it difficult for some students to access. As mentioned above, the office and counseling spaces within the Counseling Center are separated only by a partition. While suburban campuses often provide private counseling areas and lounges for users within the lecture building, this is difficult at our university, located in the city center^42, 43)^. Furthermore, with only one entrance and exit, emergency evacuation is difficult, often resulting in feelings of psychological pressure. We have repeatedly discussed this issue with the university but have not yet arrived at a solution.

Third, we must address the needs of international students. Our university has a large international student population, and some students struggle to express their psychological symptoms fluently in Japanese; this is compounded by the fact that many international students are struggling to adapt to life in a different culture. However, we also find it difficult to provide support in foreign languages, and at present, we recommend that students seek counseling in their native language at an external institution. In the future, we plan to work with faculty members on campus to address this issue.

## Conclusions

This review provides an overview of the Student Counseling Center on the Hongo and Ochanomizu campuses, which were established in October 2019, and identifies key areas for future improvement. Although fewer than 10 years have passed since its establishment, the Counseling Center represents a relatively new university-based mental health service. As student needs continue to diversify alongside Japan’s broader movement toward a more inclusive society, it is essential to further enhance accessibility, responsiveness, and cultural sensitivity within counseling services. We believe that, by collaborating with relevant parties, including other campuses within the university, and by developing a deeper understanding of these issues, we will be able to provide more student-centered support. Furthermore, we believe that collaboration with other universities in Japan will allow us to further develop the experience and knowledge that may be difficult to gain through activities conducted solely within our university.

## Author contributions

TY, YS, KT, and ST interviewed the students. TY wrote and revised the first draft. YS added figures, and KN further revised and added to the manuscript. All authors contributed to and approved the final version of the manuscript.

## Conflicts of interest statement

The authors declare that there are no conflicts of interest.

## Data availability statement

The datasets generated and analyzed during the current study are not publicly available because disclosure of personal information was not included in the research protocol.

## Ethics approval statement

This study adhered to the Ethical Guidelines for Medical Research for Humans and the Declaration of Helsinki. The research protocol was approved by the Juntendo University School of Medicine Ethics Committee (Approval No. E25-0274).

## Patient consent statement

Because this was a retrospective study that dealt with existing medical records, the requirement for informed consent was waived. Information about the study design was posted on the Juntendo University website, and all participants were given the opportunity to refuse participation.
